# ECRG4 Represses Cell Proliferation and Invasiveness via NFIC/OGN/NF-κB Signaling Pathway in Bladder Cancer

**DOI:** 10.3389/fgene.2020.00846

**Published:** 2020-08-14

**Authors:** Xin Liang, Jiangang Gao, Quan Wang, Sichuan Hou, Changli Wu

**Affiliations:** ^1^Department of Urology, The Second Hospital of Tianjin Medical University, Tianjin, China; ^2^Department of Urology, Qingdao Municipal Hospital, Qingdao, China; ^3^Department of Anesthesiology, Qingdao Municipal Hospital, Qingdao, China

**Keywords:** ECRG4, bladder cancer, OGN, NFIC, NF-κB signaling pathway

## Abstract

Bladder cancer (BCa) is a malignant tumor in the urinary system with high cancer-related mortality worldwide. However, the molecular mechanisms of many genes dysregulated in BCa are still unclear. Herein, we showed that esophageal cancer-related gene-4 (ECRG4), which is downregulated in BCa tissues and cell lines, has a positive correlation with osteoglycin (OGN). Further functional experimental studies suggested that both ECRG4 and OGN inhibit cell proliferation, migration, and invasion in BCa cells. Moreover, ECRG4 acts as a tumor repressor and promotes the expression of OGN via the upregulation of nuclear factor 1 C-type (NFIC), which can bind to the promoter region of OGN and regulate its transcription. Bioinformatics analysis revealed that NFIC is downregulated in BCa tissues and has a positive correlation with ECRG4 or OGN. Esophageal cancer-related gene-4 could positively regulate the protein levels of NFIC in BCa cells. In addition, we demonstrated for the first time that ECRG4 inhibits the nuclear factor (NF)-κB signaling pathway via the upregulation of OGN in BCa cells. Overall, these findings provide evidence that both ECRG4 and OGN function as tumor repressors and that overexpression of ECRG4 inhibits the NF-κB signaling pathway by promoting NFIC/OGN signaling in BCa cells. Our results reveal the molecular regulatory mechanisms of the ECRG4-mediated repression of the NFIC/OGN/NF-κB signaling pathway in BCa and provide potential biomarkers or therapeutic targets for BCa.

## Introduction

Bladder cancer (BCa), a malignant tumor in the urinary system, is one of the main reasons of cancer-related mortality worldwide, with 549,393 newly diagnosed cases and 199,922 deaths in 2018 (Bray et al., 2018; Siegel et al., 2018; Ferlay et al., 2019). Although considerable efforts, such as endoscopic resection and adjuvant chemotherapies, have been undertaken for BCa therapy in recent years, the therapeutic efficacy of patients with BCa remains ineffective owing to the limited effect of therapies on advanced BCa (Alfred Witjes et al., 2017). Therefore, a better understanding of the molecular mechanism of BCa progression is critical to discover novel biomarkers or treatment strategies for BCa.

Esophageal cancer-related gene-4 (ECRG4 or C2orf40) has been characterized as a tumor suppressor gene in several types of cancers, such as colorectal cancer (Gotze et al., 2009; Cai et al., 2016), esophageal squamous cell carcinoma (ESCC; Mori et al., 2007; Li et al., 2011; Wen and Hu, 2015), breast cancer (Sabatier et al., 2011; Lu et al., 2013; You et al., 2016; Tang et al., 2019), renal cell carcinoma (Luo et al., 2016), gastric cancer (Deng et al., 2018), and nasopharyngeal carcinoma (Chen J.Y. et al., 2017). Although many studies have strongly suggested the role of ECRG4 as a potential tumor suppressor gene, some other reports have also demonstrated the oncogenic role of ECRG4 in patients with papillary thyroid carcinoma (Chen J. et al., 2015) and hepatocellular carcinoma (Ge et al., 2017).

To date, many studies have focused on the upstream epigenetic regulation of ECRG4 and the promoter region of ECRG4, which is frequently hypermethylated in cancers, including BCa (Rose et al., 2020). However, few studies have been reported regarding the downstream regulation mechanism of ECRG4. Furthermore, the downstream regulation mechanisms of ECRG4 in cancers, particularly BCa, remain unclear, although Huang et al. (2019) have demonstrated that ECRG4 inhibits the migration and proliferation of oral squamous cell carcinoma via a non-coding RNA-related mechanism by repressing the BC200 lncRNA/MMPs axis.

In this study, we aimed to investigate the expression, function, and downstream molecular mechanism of ECRG4 in patients with BCa. We found that ECRG4 and osteoglycin (OGN) were both downregulated in BCa tissues and cell lines. Systematically, we demonstrated that both ECRG4 and OGN act as tumor suppressor genes and have a positive correlation in patients with BCa and healthy people. Furthermore, we demonstrated that ECRG4 inhibits cell proliferation and invasion via the nuclear factor 1 C-type (NFIC)/OGN/nuclear factor (NF)-κB axis in BCa. Our results indicate that ECRG4 and OGN may be potential biomarkers or therapeutic targets for BCa.

## Materials and Methods

### Cell Culture and Transfection

The human BCa cell lines J82, BC-5367, UMUC3, BIU-87, and T24 and human non-cancerous immortalized urothelial cell line SV-HUC-1 were purchased from ATCC and cultured in Dulbecco’s modified Eagle’s medium (DMEM; HyClone, United States) containing 10% fetal bovine serum (FBS; HyClone, United States) and 1% penicillin/streptomycin. The cells were incubated in a humid atmosphere with 5% CO_2_ at 37°C.

Cell transfection was performed using the Lipofectamine 2000^TM^ reagent (Invitrogen, United States) according to the manufacturer’s instructions.

### Vector Construction

The gene encoding ECGR4 and OGN were amplified from the cDNA of J82 cells, and the DNA fragments were cloned into a pcDNA3 vector. The shRNAs for ECGR4 and OGN knockdown were synthesized from GENERALBIOL (Anhui, China) and were annealed and cloned into a pSilencer 2.1-neo vector (Ambion). All the primers for polymerase chain reaction (PCR) amplification and all the oligonucleotides for annealing are listed in Supplementary Table S1.

### RNA Extraction and Quantitative Reverse Transcription Polymerase Chain Reaction

Bladder cancer cells were transfected with relative plasmids. After transfection for 36 h, total RNA was extracted from BCa cells using the TaKaRa MiniBEST Universal RNA Extraction Kit (TaKaRa, China). The HiScript^®^ II One Step qRT-PCR SYBR Green Kit (Vazyme, China) was used to detect the mRNA levels of ECRG4, OGN, and NFIC using the ABI7500 system (Applied Biosystems) as per the manufacturer’s instructions. The mRNA levels were quantified using the housekeeping gene GAPDH as a reference. The 2^–ΔΔCT^ method was used to evaluate the data. Primers are listed as follows (5’–3’): ECRG4-F: ACTAAGACTAAAGTGGCCGTTG, ECRG4-R: AATTTCGCTTCGTCAAAGCCC; OGN-F: TCTACACTTCTCCTGTTACTGCT, OGN-R: GAGGTAATGGTGTTATTGCCTCA; NFIC-F: ACCTGGCATACGACCTGAAC, NFIC-R: TCCATCGAGCCCGATTTGTG; and GAPDH-F: CTGGGCTACACTGAGCACC, GAPDH-R: AAGTGGTCGTTGAGGGCAATG.

### 3-[4,5-Dimethylthiazol-2-yl]-2,5 Diphenyltetrazolium Bromide (MTT) Assay

The cell viability of BCa cells (J82 and BC-5367) was detected using the MTT Assay Kit (ab211091; Thermo Fisher Scientific, United States). Briefly, BCa cells were grown on 12-well plates and transfected with relative plasmids. After transfection for 24 h, cells were seeded into 96-well plates (2 × 10^3^ cells/well) for growth. Then, after transfection for 48, 72, and 96 h, 20 μl of MTT solution was added to the culture and incubated for 4 h at 37°C. After discarding the supernatant, 100 μl of dimethyl sulfoxide was added to each well to dissolve the formazan. The absorbance value was measured at 570 nm using a spectrophotometer.

### Colony Formation Assay

The colony formation assay was performed to detect the proliferative ability of BCa cells. After transfection, the cells (300 cells/well) were seeded into 12-well plates and cultured for 14 days. Then, the medium was discarded, and 500 μl of 4% paraformaldehyde was added to each well, followed by staining with 0.1% crystal violet. The number of colonies (>50 cells) was calculated. The average number was used to evaluate the formation ability.

### Transwell Assay

The transwell migration and invasion assay was used to measure the migration capability and invasion ability of BCa cells. Briefly, BCa cells (6 × 10^4^ cells/well for migration and 8 × 10^4^ cells/well for invasion) were seeded on the top of the chamber without Matrigel for detecting migration capability and on the top of the chamber with Matrigel for invasion efficiency. To the bottom of the 24-well plate, 600 μl of the DMEM containing 20% FBS was added, and the cells were grown for 48 h. Then, the cells on the top membrane were removed and fixed on the lower chamber membrane using 4% (w/v) paraformaldehyde. Staining of the invaded cells was performed using 0.4% (w/v) crystal violet.

### Luciferase Assay

For promoter activity analysis, various lengths of human OGN gene promoter regions (-989/++91, -662/+91, and -287/+91 bp) were synthesized by GENEWIZ Biotech (Suzhou, China) and inserted into the KpnI and XhoI sites of pGL3-Basic (Promega). To perform NF-κB activity analysis, NF-κB luciferase reporter plasmid pGMNF-K B-Lu was obtained from Genomeditech (Shanghai, China). For luciferase assays, the OGN gene promoter reporter plasmids (2 μg) or NF-κB luciferase reporter plasmid pGMNF-κB-Lu was co-transfected with pRL-null (0.5 ng; Promega) into BCa cells. Luciferase activity was determined according to the ratio of firefly luciferase intensity/Renilla luciferase intensity.

### Chromatin Immunoprecipitation Assay

The chromatin immunoprecipitation assay (ChIP) assay was performed using the Chromatin Immunoprecipitation (ChIP) Assay Kit (17-295; Merck KGaA, Germany) as per the manufacturer’s instructions. Briefly, the cells were fixed with formaldehyde (1% final concentration) and incubated for 10 min at 37°C. Then, the medium was removed, and the cells were washed twice with cold phosphate-buffered saline containing protease inhibitors (1 mM phenylmethylsulfonyl fluoride). The pellet cells were lysed using the SDS Lysis Buffer and incubated for 10 min on ice. The DNA was sheared to 200–1000-bp lengths via sonication under ice-cold condition. The anti-NFIC (ab245597, 1:50; Abcam, United States) antibody was added to the fragmented chromatin mixture and incubated overnight at 4°C. Then, protein A agarose/salmon sperm DNA was added for 1 h at 4°C with rotation to collect the antibody/chromatin complex. Recovery of DNA fragments was performed according to the manufacturer’s protocol. The DNA fragments were detected via standard PCR or quantitative polymerase chain reaction (qPCR) using the specific primers for the OGN (575 bp) promoter region (5’–3’): forward: AGCACCAAGGAGGAATTTTTTAAAAGTGAC and reverse: CTGCTTCAACTAATTTGACTATAAACTTGT.

### Western Blot

Proteins were extracted using a RIPA lysis buffer, separated using a 10–12% SDS-PAGE gel, and transferred onto polyvinylidene fluoride membranes. The proteins on the membranes were probed using specific primary antibodies for ECRG4 (Cat #PA5-104506, 1:1,000; Thermo Fisher Scientific, United States), OGN (Cat #12755-1-AP, 1:2,000; Thermo Fisher Scientific, United States), β-actin (Cat #PA1-16889, 1:2,000; Thermo Fisher Scientific, United States), NFIC (ab245597, 1:500; Abcam, USA), p65 (Cat #51-0500, 1:2,000; Thermo Fisher Scientific, United States), and p-p65 (S536) (Cat #MA5-15160, 1:1,000; Thermo Fisher Scientific, United States). Horseradish peroxidase-labeled goat anti-rabbit IgG (H + L) secondary antibody (Cat # 65-6120, 1:5,000, Thermo Fisher Scientific, United States) was used as the secondary antibody, and bands were visualized using the enhanced chemiluminescence assay (Wanei Bio, China) and quantified using the ImageJ software.

### Statistical Analysis

All data are presented as mean ± SD and repeated at least three times. The difference between the groups was calculated using Student’s *t*-test or one-way ANOVA with SPSS 17.0 software (SPSS Inc., Chicago, IL, United States). A *p*-value < 0.05 was considered statistically significant.

## Results

### Downregulation of ECRG4 and OGN Expression in BCa Tissues and Cell Lines

**FIGURE 1:**
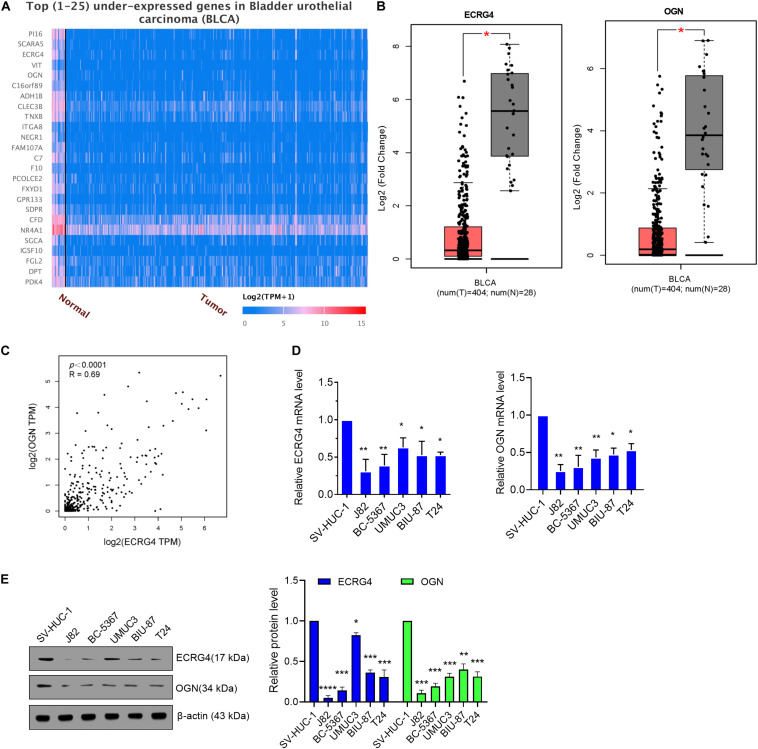
ECRG4 and OGN are downregulated in BCa cells. **(A)** Heatmap of the top 25 underexpressed genes in BCa were analyzed via the UALCAN (http://ualcan.path.uab.edu/index.html) online website. **(B)** ECRG4 and OGN mRNA levels in the GEPIA database (http://gepia.cancer-pku.cn/). **(C)** Correlation analysis of OGN and ECRG4 expression was performed using GEPIA in patients with BCa and healthy people. **(D)** Quantitative reverse transcription polymerase chain reaction (RT-qPCR) was performed to detect ECRG4 and OGN mRNA levels in BCa cell lines and in a non-cancerous immortalized urothelial cell line SV-Huc-1. **(E)** Western blotting detected the expression levels of ECRG4 and OGN in BCa cell lines and in a non-cancerous immortalized urothelial cell line SV-Huc-1. **(B)** Student’s *t*-test; **(D,E)** ANOVA followed by Bonferroni’s *post hoc* test. All data were replicated three times. *p < 0.05, **p < 0.01, ***p < 0.001.

First, we analyzed the dysregulated genes in BCa via an online website tool UALCAN. A heatmap of the top 25 underexpressed genes showed that ECRG4 and OGN were remarkably downregulated in BCa tissues compared with normal tissues (Figure 1A). Then, GEPIA was performed to verify that ECRG4 and OGN expression levels were lower in BCa tissues than in normal tissues (Figure 1B). We analyzed the correlation between ECRG4 and OGN using GEPIA in patients with BCa and healthy people. As described in Figure 1C, a positive correlation was observed between ECRG4 and OGN expression. We further confirmed the expression of ECRG4 and OGN in BCa cell lines and a non-cancerous immortalized urothelial cell line SV-Huc-1. As shown in Figure 1D, the mRNA levels of ECRG4 and OGN in BCa cell lines were downregulated compared with those in the SV-Huc-1 cell line. The mRNA levels of ECRG4 and OGN were considerably lower in J82 and BC-5367 cells than in other BCa cells. In addition, the protein levels of ECRG4 and OGN in BCa cells and SV-Huc-1 were detected, and the results showed that ECRG4 and OGN were underexpressed frequently in BCa cells compared with those in the SV-Huc-1 cell line (Figure 1E). Taken together, these results indicated that ECRG4 and OGN were both downregulated in BCa.

### ECRG4 Functions as a Tumor Suppressor Gene in BCa Cells

**FIGURE 2:**
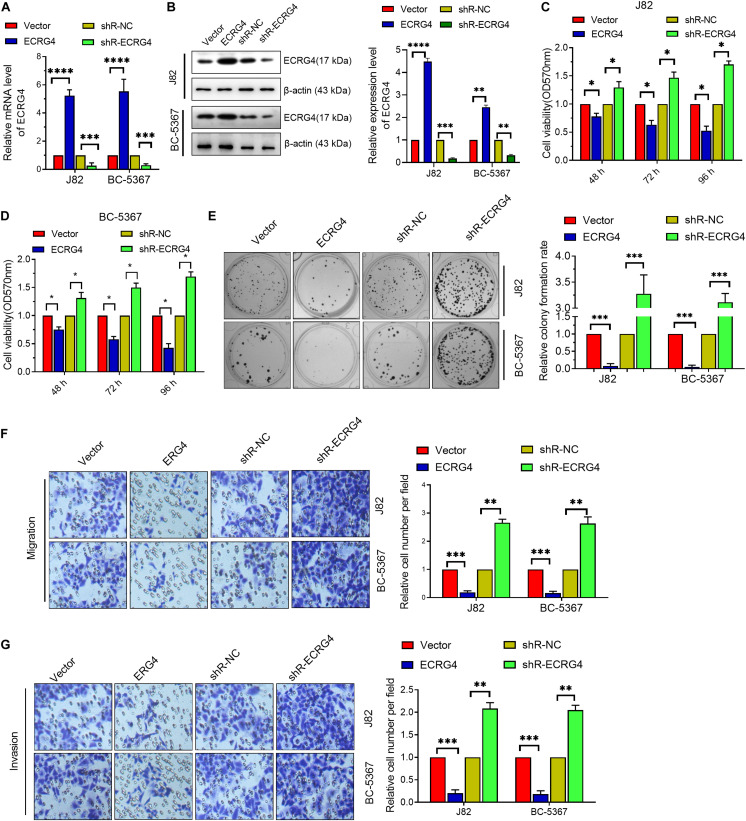
ECRG4 inhibits the proliferation, migration, and invasion of BCa cells. **(A)** RT-qPCR was performed to detect ECRG4 mRNA levels in BCa cells after transfection with ECRG4-overexpressing or silencing plasmids. **(B)** Western blotting detected the expression levels of ECRG4. **(C,D)** MTT assay was performed to detect cell viability. **(E)** The colony formation assay was performed to detect cell proliferation. **(F,G)** The transwell assay was performed to detect the effect of ECRG4 on cell migration and invasion (magnification, ×200). **(A–G)** ANOVA followed by Bonferroni’s *post hoc* test. All data were replicated three times. *p < 0.05, **p < 0.01, ***p < 0.001, ****p < 0.0001.

To investigate the role of ECRG4 in BCa cells, we first introduced exogenous ECRG4 into BCa cells or silenced the endogenous ECRG4 by transfecting ECRG4-overexpressing or knockdown plasmids. Using qPCR and Western blot, we verified that the mRNA and protein levels of ECRG4 were significantly increased in ECRG4-overexpressing cells, whereas a reverse trend was observed in ECRG4-knockdown cells (Figures 2A,B). Then, using the MTT assay (Figures 2C,D), we found that the forced expression of ECRG4 remarkably inhibited the cell viability and that the reduced expression of endogenous ECRG4 increased the cell viability. The colony formation assay results showed that ECRG4 overexpression reduced the colony formation ability of the cells, whereas ECRG4 knockdown had an opposite effect (Figure 2E), which confirmed that ECRG4 affects cell migration and invasion in BCa cells. The results demonstrate that ECRG4 upregulation leads to a remarkable reduction in cell migration and invasion, whereas ECRG4 knockout enhances the cell migration and invasion ability of J82 and BC-5367 cells (Figures 2F,G).

### OGN Functions as a Tumor Suppressor Gene in BCa Cells

**FIGURE 3:**
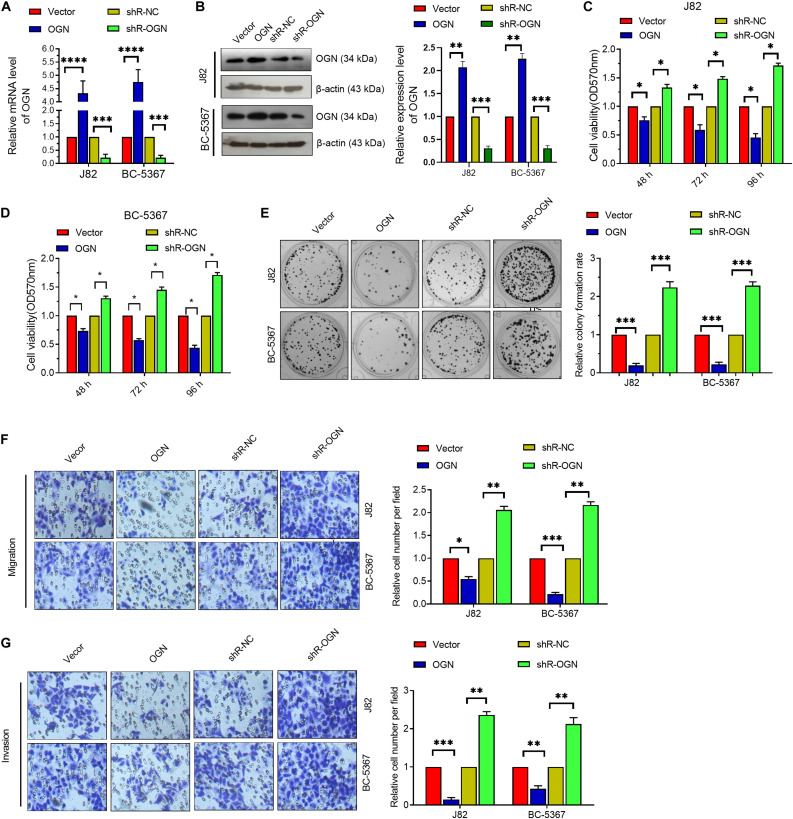
OGN inhibits the proliferation, migration, and invasion of BC cells. **(A)** RT-qPCR was performed to detect OGN mRNA levels in BCa cells after transfection with ECRG4-overexpressing or silencing plasmids. **(B)** Western blotting detected the expression levels of OGN. **(C,D)** MTT assay was performed to detect cell viability. **(E)** The colony formation assay was performed to detect cell proliferation. **(F,G)** The transwell assay was performed to detect the effect of OGN on cell migration and invasion (magnification, ×200). **(A–G)** ANOVA followed by Bonferroni’s *post hoc* test. All data were replicated three times. *p < 0.05, **p < 0.01, ***p < 0.001, ****p < 0.0001.

To verify the role of OGN in BCa cells, we first confirmed the validity of the OGN-overexpressing or knockdown plasmids via qPCR and Western blot (Figures 3A,B). Then, we explored whether OGN regulates BCa cell proliferation, migration, and invasion via the MTT, colony formation, and transwell assays. The results showed that OGN overexpression reduced the cell viability, colony formation ability, migration ability, and invasion ability of the cells, whereas OGN knockdown exhibited an opposite effect (Figures 3C–G). These collective results indicate the tumor repressor role of OGN in BCa cells.

### ECRG4 Acts as a Tumor Repressor Gene by Upregulating OGN in BCa Cells

**FIGURE 4:**
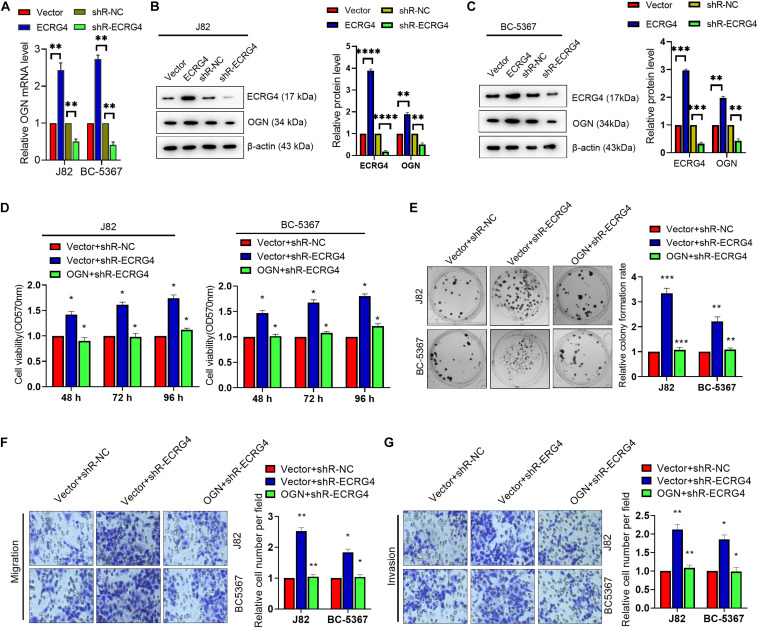
ECRG4 inhibited cell proliferation, migration, and invasion via the upregulation of OGN in the BCa cells. **(A)** The RT-qPCR and **(B,C)** Western blot detected the mRNA or protein levels of ECRG4 and OGN in the BCa cells after transfection with the ECRG4 overexpression or silencing plasmids. **(D)** The MTT assay, **(E)** the colony formation assay, and **(F,G)** transwell assay (magnification, ×200) were performed to evaluate the viability, proliferation, migration, and invasion abilities of the BCa cells. All data were replicated three times. **(A–G)** ANOVA followed by Bonferroni’s *post hoc* test. *p < 0.05, **p < 0.01, ***p < 0.001, ****p < 0.0001.

Given the consistent role of ECRG4 in BCa cells, we investigated whether changes in ECRG4 expression affect the expression levels of OGN. We observed that ECRG4 overexpression enhanced the mRNA and protein levels of OGN. On the other hand, ECRG4 knockdown reduced the mRNA and protein levels of OGN in BCa cells (Figures 4A–C). Additionally, ECRG4 knockdown remarkably increased the cell proliferation, migration, and invasion abilities of BCa cells, whereas OGN overexpression could partly counteract the promoting effect caused by ECRG4 knockdown (Figures 4D–G). Collectively, these results showed that ECRG4 inhibited cell proliferation, migration, and invasion via the upregulation of OGN in the BCa cells.

### ECRG4 Increases OGN Expression by Enhancing NFIC Levels in BCa Cells

**FIGURE 5:**
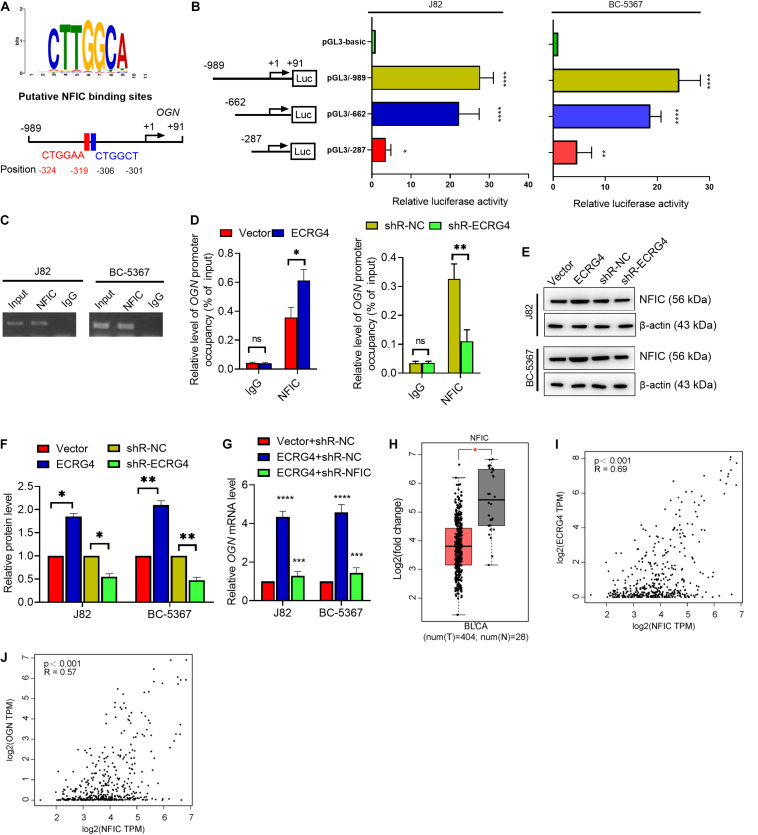
ECRG4 upregulates OGN via an NFIC-mediated mechanism in BCa cells. **(A)** The NFIC transcription factor was predicted at -989 to +91 bp of the OGN promoter region using the JASPAR online tool (http://jaspardev.genereg.net/). **(B)** A schematic representation of the truncated luciferase reporter plasmids with OGN promoter activity. **(C)** The ChIP assay was performed using the NFIC antibody. **(D)** ChIP-qPCR detected the promoter occupancy of OGN. **(E)** Western blotting detected the protein levels of NFIC in BCa cells after transfection with the indicated plasmids. **(F)** Quantification of the protein levels of NFIC in **(E)**. **(G)** RT-qPCR detected the mRNA levels of OGN. **(H)** NFIC mRNA levels in the GEPIA database. **(I)** Correlation analysis of ECRG4 and NFIC expression was performed using GEPIA in patients with BCa and healthy people. **(J)** Correlation analysis of OGN and NFIC expression was performed using GEPIA in patients with BCa and healthy people. **(B,D,F,G)** ANOVA followed by Bonferroni’s *post hoc* test; **(H)** Student’s *t*-test. All data were replicated three times. *p < 0.05, **p < 0.01, ***p < 0.001, ****p < 0.0001; ns, not significant.

To further investigate the mechanism of ECRG4-mediated upregulation of OGN in BCa cells, we analyzed the promoter region of OGN using TFBIND^1^ and predicted five potential transcription factors of OGN: HOXD10, GATA4, EBF1, TCF3, and NFIC. Knockdown of these factors showed that NFIC displayed the most potent regulatory effect on OGN transcription in J82 cells based on a quantitative reverse transcription polymerase chain reaction (RT-qPCR) assay (Supplementary Figure S1). We further found two conserved NFIC transcription factor binding sites at the -989 to +91 bp region using the JASPAR online tool (Figure 5A). Then, we determined the promoter activity of OGN by constructing a series of reporter plasmids carrying different lengths of the 5’ region of the OGN promoter. The results showed that luciferase activity at the -989/+91 region was significantly higher than other truncations in the J82 and BC-5367 cell lines (Figure 5B). The ChIP assay targeting the OGN promoter region was performed using the anti-NFIC antibody. The results showed that NFIC could bind to the OGN promoter region (Figure 5C). Furthermore, the ChIP-qPCR results showed that ECRG4 overexpression increased the binding ability of NFIC to the OGN promoter region, which was reduced after ECRG4 knockdown (Figure 5D). Additionally, we showed that ECRG4 could positively regulate the protein levels of NFIC in BCa cells (Figures 5E,F). We also found that NFIC downregulation could partly counteract the OGN mRNA levels induced by ECRG4 overexpression (Figure 5G). In addition, we analyzed the mRNA levels of NFIC using GEPIA and found that NFIC was downregulated in BCa tissues compared with normal tissues (Figure 5H). We thus analyzed the correlation between ECRG4 and NFIC or between OGN and NFIC using GEPIA in patients with BCa and healthy people. As shown in Figures 5I,J, we found that the expression between ECRG4 and NFIC or between OGN and NFIC exhibited significant positive correlations, which are consistent with the result that ECRG4 overexpression promotes OGN expression via a NFIC-mediated mechanism in BCa cells.

### ECRG4 Represses NF-κB Pathway by Increasing OGN Expression in BCa Cells

**FIGURE 6:**
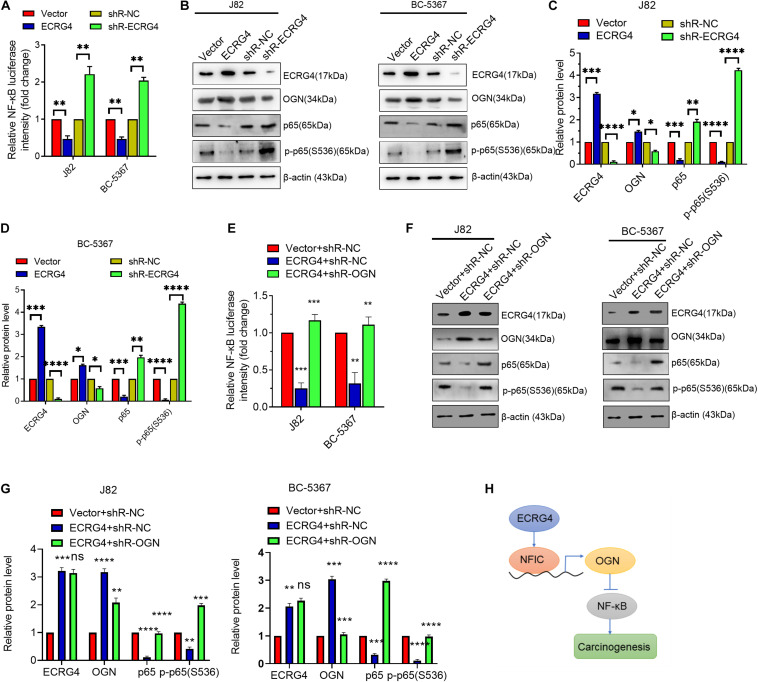
ECRG4 inhibits the NF-κB pathway via the upregulation of OGN in BCa cells. **(A)** NF-κB transcriptional activity was detected using an NF-κB luciferase reporter gene system. **(B)** Western blotting detected the protein levels of ECRG4, OGN, p65, and p-p65 (S536) in BCa cells after transfection with ECRG4-overexpressing or silencing plasmids. **(C,D)** Quantification of the protein levels of ECRG4, OGN, p65, and p-p65 (S536) in panel **(B)**. **(E)** The NF-κB luciferase reporter gene system was used to detect the NF-κB transcriptional activity of BCa cells after transfection with indicated plasmids. **(F)** Western blotting detected the protein levels of p65 and p-p65 (S536) in BCa cells after transfection with the indicated plasmids. **(G)** Quantification of the protein levels of p65 and p-p65 (S536) in panel **(F)**. **(H)** The proposed mechanism of ECRG4 inhibition of the carcinogenesis of BCa cells via NFIC/OGN/NF-κB signaling. **(A–G)** ANOVA followed by Bonferroni’s *post hoc* test. All data were replicated three times. *p < 0.05, **p < 0.01, ***p < 0.001, ****p < 0.0001.

To further explore the downstream mechanism of ECRG4 in BCa cells, we examined whether ECRG4 regulates the NF-κB signaling pathway, which has been reported to play an important role in several types of cancers (Hoesel and Schmid, 2013; Liu et al., 2018; Yang et al., 2019; Li et al., 2020). We first determined the effect of ECRG4 expression on the NF-κB transcriptional activity using an NF-κB luciferase reporter gene system. We observed that ECRG4 overexpression decreased NF-κB activity, whereas ECRG4 knockdown increased NF-κB activity (Figure 6A). Ectopic expression of ECRG4 resulted in the inhibition of the total p65 and p-p65 (S536) subunit, whereas ECRG4 knockdown enhanced the total p65 and active form of p-p65 (S536) (Figures 6B–D). Furthermore, the luciferase assay indicated that OGN knockdown resulted in an increase in NF-κB activity, which was repressed by ECRG4 overexpression and has been confirmed by Western blotting (Figures 6E–G). Taken together, these results suggest that ECRG4 inhibits the carcinogenesis of BCa cells by inhibiting the NF-κB signaling pathway, which was mediated by OGN.

## Discussion

Increasing evidence has shown that dysregulated genes play vital roles in cancer progression (Fang et al., 2013). In this study, three main complementary findings were highlighted: (1) there is a positive correlation between ECRG4 and OGN in BCa cells, (2) ECRG4 acts as a tumor repressor and promotes OGN expression via a NFIC-mediated mechanism, and (3) repression of the ECRG4/NFIC/OGN axis activates the NF-κB signaling pathway in BCa cells. A novel regulation mechanism is proposed in Figure 6H, in which ECRG4 promotes OGN expression via the upregulation of NFIC, the nuclear factor I (NFI) family of site-specific transcription factors in the transcriptional modulation of various gene expressions. Thus, ECRG4 impedes the activation of downstream NF-κB signaling via the OGN-mediated mechanism in BCa cells.

Presumably, the current study was the first to find a positive correlation between ECRG4 and OGN in BCa cells. A study has reported that ECRG4 is downregulated and acts as a tumor suppressor gene in most types of cancers (Qin and Zhang, 2019). Several studies have shown that the ECRG4 promoter is frequently hypermethylated in patients with breast cancer, gastric cancer, and nasopharyngeal carcinoma, resulting in the downregulation of ECRG4 (You et al., 2015; Deng et al., 2018; Tang et al., 2019). Consistent with a previous study that reported that ECRG4 is hypermethylated in BCa cells (Rose et al., 2020), our results further demonstrated that ECRG4 is significantly downregulated in BCa tissues and cell lines. Furthermore, we observed that ECRG4 expression was positively correlated with OGN, which is a tumor suppressor in colorectal cancer and breast cancer (Hu et al., 2018; Xu et al., 2019). Consistent with a previous study, we also found that OGN is downregulated in BCa tissues and cell lines. Further functional and experimental studies suggest that both ECRG4 and OGN inhibit cell proliferation, migration, and invasion in BCa cells.

To further verify the relationship between ECRG4 and OGN, we showed that ECRG4 overexpression can increase the mRNA and protein levels of OGN, whereas ECRG4 knockdown can inhibit mRNA and protein levels, suggesting that ECRG4 plays an essential role in BCa by regulating OGN expression. Indeed, we demonstrated that the repression of endogenous ECRG4 could increase the cell proliferation, migration, and invasion abilities of BCa cells; conversely, co-transfection with OGN-overexpressing and ECRG4-knockdown plasmids partly counteracted the promoting effect caused by ECRG4 downregulation, suggesting that ECRG4 inhibits cell proliferation, migration, and invasion by upregulating OGN in BCa cells. To further explain the mechanism for the ECRG4-mediated upregulation of OGN in BCa cells, the NFIC transcription factor was predicted at the promoter region of OGN using the JASPAR online tool. Based on the results from the promoter activity assay, ChIP assay, and ChIP-qPCR assay, we preliminarily observed that NFIC could bind to the OGN promoter region via ECRG4 regulation, further confirming that ECRG4 can positively regulate the protein levels of NFIC in BCa cells. Bioinformatics analysis showed that NFIC was downregulated in BCa tissues and had a positive correlation with ECRG4 or OGN, suggesting that ECRG4 overexpression promotes OGN expression via the NFIC-mediated mechanism in BCa cells. Presumably, NFIC, an important member of the NFI family (NFIA, NFIB, NFIC, and NFIX) that exhibits a highly conserved DNA-binding domain at their N-termini, has shown to have transcriptional repression and activation potentials (Chen K.S. et al., 2017). A previous study has suggested that the NFIC-mediated repression of the MMTV promoter needs interaction with coactivators p300/CBP and SRC-1 (Chaudhry et al., 1999). Eeckhoute et al. (2006) illustrated that NFIC induction could directly repress CCND1 transcription by dissociating estradiol stimulation in breast cancer. However, Lee et al. (2015) showed that NFIC could directly bind to the promoter region of KLF4 and increase its transcriptional activity in breast cancer. Although several studies have shown that NFIC proteins could have repression and activation potentials, the underlying mechanism of transcription regulation remains unclear.

A previous study has suggested that ECRG4 overexpression represses NF-κBp65 expression and nuclear translocation in ESCC cells (Li et al., 2009); however, its potential mechanism is still unclear. Herein, downregulation of total p65 by ECGR4 knockdown suggests that ECGR4 inhibits NF-κB activity by decreasing p65 expression. Moreover, we first demonstrated that ECRG4 overexpression inhibits the NF-κB signaling pathway by OGN upregulation in BCa cells. To date, rarely reports have investigated the transcriptional regulation of OGN in its upstream promoter region. However, OGN post-transcriptional regulation by miRNAs, such as miR-155 and miR-1305, has been demonstrated recently (Freire et al., 2017; de Oliveira et al., 2018; Wen et al., 2020). In addition, the downstream signaling pathways of OGN have been well investigated. For example, Xu et al. (2019) have reported that OGN represses PI3K/Akt/mTOR signaling in breast cancer. Hu et al. (2018) reported that OGN inhibits EMT and invasiveness via the repression of EGFR/Akt signaling in colorectal cancer. The PI3K/Akt or EGFR/Akt pathway is the upstream signaling of the NF-κB pathway. Therefore, from our results, we can conclude that ECRG4 represses the NF-κB pathway by promoting OGN expression.

In summary, this is the first demonstration of the role and molecular mechanism of ECRG4 in BCa cells. Our data suggest that ECRG4 is downregulated in BCa tissues and cells. Unprecedentedly, we have also shown that ECRG4 has a positive correlation with OGN, which is regulated via ECRG4, at least in part by the transcription factor NFIC-mediated mechanism. Esophageal cancer-related gene-4 inhibits the NF-κB signaling pathway by promoting NFIC/OGN signaling in BCa cells. Our results highlight the significance of the ECRG4-mediated NFIC/OGN/NF-κB signaling pathway in BCa cells and reveal that ECRG4 and OGN may be potential biomarkers or therapeutic targets for BCa.

## Data Availability Statement

The original contributions presented in the study are included in the article/Supplementary Material, further inquiries can be directed to the corresponding author.

## Author Contributions

CW designed and supervised the study. XL performed the study and analyzed the data. JG, QW, and SH analyzed the data. XL and CW wrote the manuscript. All authors approved the final manuscript.

## Conflict of Interest

The authors declare that the research was conducted in the absence of any commercial or financial relationships that could be construed as a potential conflict of interest.

## Footnotes

1 http://tfbind.hgc.jp/
