# Supplementary material for: ECRG4 Represses Cell Proliferation and Invasiveness via NFIC/OGN/NF-κB Signaling Pathway in Bladder Cancer
Source: Front Genet. 2020 Aug 14;11:846. doi: 10.3389/fgene.2020.00846 (PMC7456849; doi:10.3389/fgene.2020.00846)
Supplement: Supplementary file 1 [file Data_Sheet_1.PDF]

Supplementary Table S1. Primers for plasmid construction in this study

Name	Sequence (5’-3’)
EGRG4-forward	CCCAAGCTTATGGCTGCCTCCCCCGCGCGGCCT
EGRG4-reverse	CCGCTCGAGTTAGTAGTCATCGTAGTTGACGCTGG
OGN-forward	CCCAAGCTTATGAAGACTCTGCAGTCTACACTTCTCC
OGN-forward	CCGCTCGAGTTAAAAGTATGACCCTATCGGTAATCTT
shR-NC	CTGGGAGGTGGATGTTTATTCCTCGAGGAATAAACATCCACCTCCCAG
shR-EGRG4	GAGATCGAAATGGACATGAATCTCGAGATTCATGTCCATTTCGATCTC
shR-OGN	GCTTCAATTACAGATGACACACTCGAGTGTGTCATCTGTAATTGAAGC
