# Supplementary material for: ECRG4 Represses Cell Proliferation and Invasiveness via NFIC/OGN/NF-κB Signaling Pathway in Bladder Cancer
Source: Front Genet. 2020 Aug 14;11:846. doi: 10.3389/fgene.2020.00846 (PMC7456849; doi:10.3389/fgene.2020.00846)
Supplement: Supplementary file 2 [file Data_Sheet_2.PDF]

Supplementary Figure S1

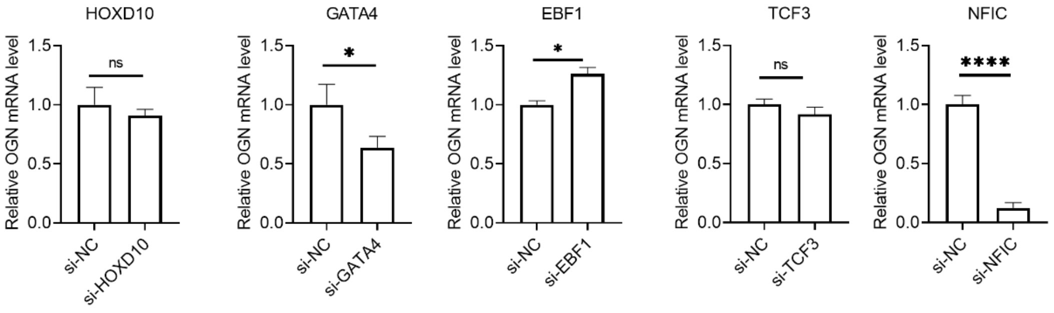

Figure S1. RT-qPCR detected the mRNA levels of OGN after silencing of 5 hypothetic transcription factors in J82 cell. *Student's t-test*. All data were replicated three times. *$p < 0.05$; ****$p < 0.0001$; ns, not significant.
